# A normative study for photographs of celebrities in Spain

**DOI:** 10.1371/journal.pone.0197554

**Published:** 2018-05-16

**Authors:** Alejandra Marful, Antonio M. Díez-Álamo, Susana Plaza-Navas, Angel Fernandez

**Affiliations:** 1 Departamento de Psicología, Universidad de Jaén, Jaén, Spain; 2 Centro de Investigación Mente, Cerebro, y Comportamiento (CIMCYC), Universidad de Granada, Granada, Spain; 3 Departamento de Psicología Básica, Psicobiología y Metodología de las Ciencias del Comportamiento, Universidad de Salamanca, Salamanca, Spain; 4 Instituto de Integración en la Comunidad (INICO), Universidad de Salamanca, Salamanca, Spain; University of Melbourne, AUSTRALIA

## Abstract

Research on familiar faces has recurrently been conducted in different domains, such as, psycholinguistics, memory, attention, face processing, aging studies, etc. In general, photographs of celebrities, their proper names, or their occupations have been the materials mainly employed in those types of studies. These stimuli are, however, very constrained by the geographic and sociocultural contexts in which the studies are conducted, and, in spite of their relevance for psychological research, there are no normative studies for celebrities in Spain. With the aim of filling this gap, the photographs and names of the 118 most frequently produced celebrities in Spain were collected. For each celebrity, values for 13 different indices (including psycholinguistic properties, naming times, and emotional indicators) were obtained from a young adult Spanish sample. Regression analyses on the data indicated that the main determinant in naming times and ToTs was the percentage of correct responses. Face agreement was also a significant predictor of ToTs. Results were compared with previous celebrity norms in other languages, and discussed in relation to the current models of face processing. These norms are likely to make a useful contribution to the design of more controlled research and applied tools in Psychology.

## Introduction

Normative studies are valuable tools in cognitive research, where manipulation and control of multiple variables are often required. Their relevance is noticeable when attention is paid to the increasing growth of published normative studies in recent years: if there were 21 published papers with the keyword “normative study” in the Psycinfo database between 1974 and 1984, and 31 papers between 1985 and 1995, this number increased to more than 200 papers over the last decade (i.e., 1996–2016). Norms of different kinds have recurrently been collected both in the verbal and non-verbal domains, and in different languages (e.g., [1–7]). When focused on the non-verbal domain, pictures of objects are probably the kind of stimuli most frequently studied. Thus, for example, after the seminal work of Snodgrass and Vanderwart [8] with standardized line drawings for an English-speaking population, we can find research that replicated and expanded their study for a variety of languages (in Spanish [9–11]; in English [12]; in French [13]; in Mandarin Chinese [14]; or in Russian [15]).

Photographs of familiar people (i.e., celebrities) are also a type of non-verbal material employed in psycholinguistics and other cognitive realms. However, different from pictures of objects, the collection of normative data for depictions of familiar people has been much scarcer. Thus far, only four studies have collected normative data for celebrities: Bonin, Perret, Méot, Ferrand, and Mermillod [16] in French; Rizzo, Venneri, and Papagno [17] and Bizzozero, Lucchelli, Saetti, and Spinnler [18] in Italian; and Smith-Spark, Moore, Valentine, and Sherman [19] in English. Still, and in spite of the small number of studies, norms for celebrity faces can be of great help when attempting to address some very relevant cognitive and linguistic issues, such as the generalization of speech production models. In this line, there is a current debate about whether face naming (i.e., proper name retrieval) is similar to or different from object naming (i.e., common name retrieval), since it is well known that proper name retrieval is slower and more error prone than the retrieval of common names or biographic information [20–25]. In addition, there is a controversy regarding the possibility that faces, unlike objects, are represented associatively rather than categorically ([26,27]; but see [28], or [22] for a different perspective). It is also well known that aging has traditionally been related to an impairment in proper name retrieval, with some researchers advancing the proposal that there is a disproportionate impairment in older adults when retrieving proper names but not common names (e.g., [20,29–33]; but see [34,35], for a different interpretation). Finally, from a more global perspective, different theoretical models have been proposed to explain face naming in ways that differ from object naming to a certain extent (e.g., [36–39]). For example, most accepted models of face processing (e.g., [39,40]) assume that seeing a familiar face (e.g., the photo of a celebrity) activates its stored representation at the face recognition unit (FRU) level, which contains representations of the structural features of familiar faces independently of the face position, angle, or lighting (but see a recent neural model in primates from Chang and Tsao [41], where face identity coding is not exemplar-based but depends on neurons that distinguish facial features along specific axes). The activation of an FRU spreads to the next pool, the person identity node (PIN), where the face is identified as being familiar. These PINs are token markers that are considered to be modality independent; i.e., the PIN can be activated by a familiar face, but also by its voice or its name. PINs are connected to both semantic information and name representations, which can be stored together [39] at the semantic information unit (SIU) or in separate pools [40]. Importantly, representations at the PIN level are considered to be specific to face naming, and they are not present in object naming models, where semantic activation spreads directly to the lexical level [42,43].

Given these apparent discrepancies between face naming and object naming, studying the variables that modulate face naming during the lexicalization process turns to be a relevant research topic. And in this regard, the effort to carry further normative studies on variables that are potentially crucial in face processing, extending their scope to different populations, has the potential to be of interest and importance. It should be noted that celebrity norms are highly constrained by geographic context and cultural peculiarities. Thus, although we can certainly find certain famous people that are universally known, there are also many celebrities who are only famous in particular contexts (e.g., only 2% of the celebrities that comprised the British norms of Smith- Spark et al. [19] appeared in the French norms of Bonin et al. [16]). Therefore, collecting celebrity norms for a given country or cultural niche seems to be the appropriate course of action. With that idea on mind, the present study aimed at collecting norms for celebrity faces in Spain, data that are not currently available, to the best of our knowledge.

Previous celebrity norms have been centered on the study of variables traditionally relevant in object naming [16,19] (notice that the studies developed by Rizzo et al. [17] and Bizzozero et al. [18] had mainly clinical purposes, and a dimensions of a different kind were normed, e.g., famous person recognition, semantic knowledge about the famous person). For example, Smith-Spark et al. [19] collected ratings of familiarity, distinctiveness, age of acquisition (AoA), objective surname frequency, and number of phonemes for a set of 696 celebrities with a sample of British participants over 40 years old. In the same spirit, Bonin et al. [16] selected 105 celebrities with a sample of French younger adults and collected values for distinctiveness, proper name agreement, face agreement, AoA, subjective frequency, and number of phonemes, with the further goal of using theses scores as possible predictors in naming response times (RTs) and *tip of the tongue* states (ToTs). Because the study conducted by Bonin and colleagues is relatively more recent, and because it included a larger number of variables, we aimed to replicate this study with a Spanish sample of young adults. To start, we selected celebrities in Spain and then we collected values for proper name agreement, distinctiveness, face agreement, AoA, subjective frequency, and number of phonemes. These latter scores were obtained from the *EsPal* database [44]. There were a few cases (in general, foreign proper names) where the output of the EsPal database was not consistent with the common Spanish phonetics [e.g., Hitler appeared as /itler/ (five phonemes), but the typical Spanish pronunciation uses to include an initial /x/ or /h/ (six phonemes)]. In these cases, two independent judges revised these special cases, marked with an “*” in the database using the alphabet of the *Journal of Spanish Filology* [45].

Together with the variables that have been commonly considered relevant in psycholinguistic studies, we decided to increase the value of our face norms by adding emotional ratings. It seems unquestionable that faces elicit emotional reactions, and it has been claimed that face recognition involves the retrieval of emotional aspects associated with the corresponding person [46]. Consequently, a variety of research findings on the emotional aspects associated with the processing of familiar faces have been reported focusing in aspects such as the special status of personally familiar faces [47], the physiological correlates associated to the faces of romantic partners [48] or the mechanisms underlying impairments like delusional person recognition [49]. Following well established procedures [50,51], valence, arousal, and dominance ratings for the facial stimuli were collected and included in the final database.

## Methods

The normative study was conducted in three different phases. There was a *first phase* where popular occupational categories (professions) were used as cues in exemplar generation tasks aimed at selecting professions with a reasonable number of exemplars and at identifying concrete celebrities in each of the selected professions. Photographs of the most frequently produced exemplars during the exemplar generation task were selected at this preliminary stage, for use in the remaining phases of the study. The *second phase* consisted of a proper-name agreement task where the selected photographs were presented as cues for name generation. The most frequently generated names and their correspondent photographs were employed during the *third phase*, that was the core of the normative study. During this final phase, subjective indices (distinctiveness, age of acquisition, face-name agreement, subjective frequency, emotional values) and objective indicators (naming times, ToTs, number of phonemes) were collected. The study was approved by the University of Jaen ethic committee.

### Participants

In total, 536 undergraduate students from the Universities of Jaén and Salamanca, in Spain, participated in this study. All were native Spanish speakers, received academic credit in exchange for their participation, and gave written informed consent. Participants were recruited from May, 2015 until December 2016 through sign-up forms distributed in Psychology classrooms and by Internet ads located at a virtual classroom platform. Equipment failures erased data from 27 participants (13 during the first phase and 14 in the second phase). Data from 6 participants who apparently did not follow the instructions (e.g., had long interruptions during their performance) and from 6 participants who were not native Spanish speakers were removed from the study. As a result, the drop-out rate for this normative study was 7.3%. There were different participants in each of the main tasks, as detailed below.

### Procedure and materials

#### First phase: Celebrity selection

A total of 92 Psychology students (79 females, mean age = 20.34, *SD* = 3.34, age range = 18–44 years old) were provided with 40 popular occupational categories for an exemplar generation task. The task was performed individually and it was controlled by the online platform *LimeSurvey* [52], with categories presented in a random order for each participant. For each occupational category, participants were instructed to write celebrity names during a 1-minute period and they were allowed to write a given name in as many categories as they thought appropriate (i.e., actor and singer). The 24 categories with the highest number of exemplars were selected to conform the final database. Although specifically arising from the current Spanish socio-demographical context, this set shared a total of 20 categories with the normed stimuli of Bonin et al. [16] and Smith-Spark et al. [19].

The 24 selected categories were presented to a new sample of 160 Psychology students (124 females, mean age = 21.03, *SD* = 4.82, age range = 17–49 years old) for an exemplar generation task in the online *LimeSurvey* platform. Two sets of 12 categories were created, so that half of the participants were presented with one set while the other half were presented with the other set. In each set, categories were randomly presented and participants produced exemplars during 1 minute. Only exemplars produced by at least 20% of the sample were selected as target stimuli for the normative database, resulting in categories that varied in size from 2 (writers) to 13 (soccer players). Colored photographs or (in a few cases) paintings of the faces of 121 selected celebrities were downloaded from images available in the Internet and edited to standardize silhouettes and background (as in [27]).

#### Second phase: Proper-name agreement task

A total of 40 Psychology students (36 females, mean age = 19.12, *SD* = 1.79, age range = 17–26 years old) participated in the proper-name agreement task. The task was conducted in groups from 1 to 16 participants and stimuli appeared electronically, using E-Prime (version 2.0, [53]), a software package also employed in the remaining phases of the study. The 121 previously selected photographs were presented in random order for a written-name generation task. Participants were instructed to write the first name that came to mind (the first name/s, the second name/s, or both). This decision was based on previous face naming studies in Spain (e.g., [22, 54]), where participants recurrently preferred a given name (e.g., *Zapatero* for the former Spanish president José Luis Rodríguez Zapatero); consequently, we adopted the instructions provided by Snodgrass and Vanderwart [8], where participants were free to produce the first name that came to mind. Notice that this procedure differs from the one used in the Bonin et al. [16] study, where participants had to write the first and second names for each celebrity. When participants were not able to produce a response, they had to indicate the reason (they did not know the famous person, they knew the famous person but not his/her name; they knew the famous person’s name, but they suffered a ToT experience) using an appropriate option in the computer’s numeric keyboard. Each trial started with a fixation point (300 ms), followed by a photograph for name generation (10 s), a blank slide (300 ms), and a slide to indicate the reason for a non-response (5 s) when appropriate.

For each photograph, the most frequent instance was selected, except when the most frequent name was ambiguous (for example *Felipe* can refer to the king of Spain or to a Spanish ex-president), erroneous (*Mozart* instead of *Beethoven*), or tied in frequency with another name. In all these cases, the most frequent exemplar (modal name) produced in the previous phase was selected. Three photographs did not produce any response and were deleted, with the result that 118 exemplars were employed in the following phases.

#### Third phase: Index Recollection

A total of 200 participants were involved in the rating tasks, following instructions that were similar to the ones used in previous studies. Participants were tested collectively, and items appeared in random order in the different tasks.

**Face-name agreement**: (40 participants, 34 females; mean age = 20.07, *SD* = 1.56, age range = 19–25 years old). In this task, participants indicated the extent to which a presented photograph corresponded to the mental image they had of the depicted celebrity. Thus, after a fixation point (500 ms), the celebrity name was printed on the center of the screen (2 s). After that, a fixation point appeared and participants were instructed to generate their mental image of the celebrity face. Finally, after 5 seconds, the photograph of the celebrity was presented and participants rated the agreement between their mental image and the presented face in a 5-point Likert scale (1-low agreement, 5-high agreement).

**Subjective frequency task**: (40 participants, 33 females; mean age = 20.47, *SD* = 2.13, age range = 19–27 years old). Participants were presented with the celebrity name and they were instructed to indicate how often they meet this celebrity (TV, internet, newspapers, etc.) in a seven-point Likert scale (1-never heard, seen or produced, 2-once a year, 3-once a month, 4-once a week, 5-every two days, 6-once every day, 7-more than once every day). After a fixation point (500 ms), each celebrity name appeared next to the Likert scale until a response was given or 9 seconds elapsed.

**Age of acquisition**: (40 participants, 31 females; mean age = 20.42, *SD* = 2.26, age range = 19–28 years old). Participants indicated the age, in years, at which they estimated they had met the famous person for the first time. Notice that this instruction is slightly different from Bonin et al.’s [16] study, where participants had to indicate the age estimation using a 7-point Likert scale. We modified the provided instructions based on recent age-of-acquisition normative studies (e.g., [55,56]) that have demonstrated the reliability and greater simplicity of the employed procedure. Each trial consisted of the presentation of a fixation point (500 ms), followed by a proper name, and participants had a maximum of 4 seconds to respond.

**Distinctiveness**: (40 participants, 28 females, mean age = 20.67, *SD* = 2.57, age range = 19–30 years old). Participants were asked to indicate if a celebrity was easily identifiable on the basis of her/his physical features. Similar to Bonin et al. [16] and Smith-Spark et al. [19], participants had to rate distinctiveness in a 7-point Likert scale (1-typical face, difficult to identify; 7-distinctive face, easy to identify) when provided with the celebrity names. Importantly, participants made their distinctiveness judgments based on their mental image of the celebrity. Each trial consisted of a fixation point (500 ms), followed by a slide with the proper name next to the Likert scale (for a maximum of 4 s).

**Affective evaluation task**: (40 participants, 35 females, mean age = 20.75, *SD* = 3.61, age range = 19–40 years old). This task was not included in any previous celebrity normative study. Here, participants provided ratings for each celebrity in the three 9-point Likert scales of the Self-Assessment Manikin (SAM; [50,51]: valence (1-happy, 9-unhappy), arousal (1-excited, 9-calm), and dominance (1-controlled, 9-in-control). Materials and procedure were close to the Bradley and Lang [50] study. Each trial consisted of a fixation point (1 s) followed by three slides with the celebrity name centered and the valence, arousal, and dominance scales on the bottom (4 s was the maximum response time per slide). Finally, a slide (500 ms) indicated the presentation of the next famous name.

**Collection of naming times**: The 118 selected photographs were presented to 44 participants (36 females, mean age = 20.11, *SD* = 1.59, age range = 18–24 years old) for a naming latency task. Each participant was tested individually. Participants were instructed to name each photograph (their first name/s, their second name/s or both) as soon as possible without errors nor hesitations. If they did not respond, they had to indicate the reason using the keyboard (they did not know the famous person; they knew the famous person but not his/her name; they knew the famous person’s name, but they had a ToT experience). Photographs were presented in a fixed random order, with breaks every 10 trials. The celebrity photograph to be named (4 s maximum) was preceded by a fixation point (500 ms), and followed by a 4 s period for the participants to indicate the reason of a non-response. Naming errors, hesitations, and voice-key failures were recorded by the experimenter.

## Results and discussion

### The dataset

The dataset is available as supplementary material. This material includes a spreadsheet file (S1 Table) in which the 118 celebrities (their modal name) are listed in alphabetic order, grouped by their occupational category (in English and Spanish). Mean scores of AoA, distinctiveness, emotional ratings (valence, arousal, and dominance), face agreement, subjective frequency, number of phonemes, two measures of name agreement [proper name agreement (PNA) for the first, second, and the complete names; and H scores for the first, second, and the complete names], percentage of correct responses, proportion of ToTs, and response times (RT) are provided. The complete set of edited photographs is available from the authors, exclusively for non-profit academic purposes.

The reliability of the different scales was evaluated employing the split-half method (see, for example, [57]). To this end, participants were randomly divided into two equal groups in each scale, and the values obtained were correlated employing the Spearman-Brown correction. In general, with a pair of exceptions that showed moderate correlations (dominance and arousal), the analyses revealed high correlation values for the rest of the variables (ranging from .73 to .97, with an average of .83), an indication of the general high reliability of the scales. See Table 1.

**Table 1:** Descriptive statistics and reliability values.

		AoA	distinctiveness	face agreement	frequency	Phonemes	PNA	H	percentage	TOT	valence	arousal	dominance
**Mean**		12.71	4.89	3.93	3.16	10.03	0.29	0.59	0.46	0.10	4.67	4.81	5.48
**Standard Deviation**		2.42	0.82	0.80	0.84	3.56	0.20	0.26	0.34	0.08	1.22	0.49	0.35
**Asymmetry**		-0.10	0.04	-1.11	0.93	0.35	0.91	0.61	0.02	1.20	0.49	-0.57	0.39
**Percentile**	**25**	11.29	4.21	3.51	2.51	7.00	0.08	0.00	0.12	0.05	3.80	4.51	5.24
	**50**	12.66	4.85	4.25	3.02	10.00	0.22	0.46	0.48	0.08	4.62	4.88	5.47
	**75**	14.29	5.58	4.56	3.66	12.25	0.42	1.02	0.78	0.15	5.30	5.16	5.61
**Reliability**		.96	.87	.95	.95	-	.95^1^	.73	.97	-^2^	.93	.51	.49

Proper name agreement (PNA), H scores, percentage of responses (percentage), tip of the tongue states (TOT), number of phonemes, age of acquisition (AoA), distinctiveness, face agreement, subjective frequency, valence, arousal, and dominance.

Notes:

^1^Given that the total sample was divided into two groups of participants, and the number of first, second, and complete name responses were expected to be low for some items, we collapsed first, second, and complete name responses to carry out proper name agreement analysis.

^2^Reliability was not calculated for ToTs due to the low number of ToT responses per item.

The validity of the norms could not be directly tested, because there are not previous celebrity norms in Spanish (and as stated before, there are almost no celebrities in common in the studies published in other languages). However, given that two different samples of participants produced celebrities from the same 24 occupational categories during the first phase, we carried out a validity analysis on these data. To recapitulate, in the first phase of the study, participants were provided with 40 occupational categories for an initial exemplar generation task, and later, the 24 categories that elicited more exemplars were selected for a second exemplar generation task. Because there was a double test of these 24 categories, using essentially the same task with two separate samples, the validity of the exemplar-generation task can be estimated by analyzing the stability of the exemplar generation task across the two samples. Although correlational analyses have traditionally been employed to test for relatedness among tasks, other statistics have been preferred when category exemplars are the focus of analyses (see for example [58], for a description of the problems related to correlation use in category norms). Therefore, we estimated the validity of the exemplar generation task using the Hellinger Affinity (HA) index [58]. HA is calculated by summing the square root of the product of the two exemplar proportions (*p*_*i*_, *q*_*i*_), and it indicates the degree of overlap between category exemplars in two separate sets of category norms, with values ranging from 1 (indicating two identical distributions) to 0 (indicating no overlap between the exemplar frequencies).

$$HA\;(p,q)=\sum_{i=1}^{n}\sqrt{p_{i}q_{i}}$$

Results indicated an overall high overlap between the two exemplar generation tasks with low variability in the observed scores (*M* = .81, *SD* = .09). In fact, as described in Table 2, HA values ranged from .61 in the TV stars category to .92 in Formula 1 drivers. In consequence, these data support the validity of the category generation task.

**Table 2:** Hellinger Affinity (HA) scores per category from the two exemplar generation tasks during the preliminary phase.

Category (Spanish)	Category (English)	HA
ACTORES/ACTRICES	ACTORS/ACTRESSES	.70
CANTANTES	SINGERS	.72
CIENTÍFICOS/AS	SCIENTISTS	.79
DEPORTISTAS	SPORTMEN/WOMEN	.81
DIRECTORES/AS DE CINE	FILM DIRECTORS	.87
DISEÑADORES/AS	FASHION DESIGNERS	.66
ENTRENADORES/AS DE FÚTBOL	SOCCER COACHES	.85
ESCRITORES/AS	WRITTERS	.73
ESTRELLAS DE TELEVISIÓN	TV STARS	.61
FAMOSOS DEL CORAZÓN	GOSSIP SHOW CELEBRITIES	.86
FUTBOLISTAS	FOOTBALLERS	.89
HUMORISTAS	COMEDIANS	.80
JUGADORES/AS DE BALONCESTO	BASKETBALL PLAYERS	.86
MIEMBROS DE LA REALEZA	ROYALTY MEMBERS	.86
MÚSICOS	MUSICIANS	.64
PERSONAJES DE CÓMIC	CARTOON CHARACTERS	.86
PERSONAJES HISTÓRICOS	HISTORICAL FIGURES	.74
PILOTOS DE FÓRMULA 1	FORMULA 1 DRIVERS	.92
PINTORES	PAINTERS	.89
POLÍTICOS/AS	POLITICIANS	.88
PRESENTADORES/AS	TV PRESENTERS	.81
PRESIDENTES/AS	PRIME MINISTERS	.87
TENISTAS	TENNIS PLAYERS	.87
TOREROS	BULLFIGHTERS	.86

### Descriptive statistics

First, descriptive statistics for the proper-name agreement task were computed (see Table 1). In general, name agreement has been located at the structural level [59], consistently with the finding that there is less agreement naming a violin than naming a piano because the first can be confused with a guitar. Therefore, Bonin et al. [16] suggested that proper name agreement should be located at the FRU level (e.g., less proper name agreement when naming Koffi Annan because his facial features can be confused with Morgan Freeman’s). Similar to Bonin et al., two indices of proper-name agreement were calculated. First, we computed the proper name agreement index (PNA), this is, the percentage of modal name responses out of the total responses for a given celebrity. We also calculated a name agreement index called H statistic [8]. H values close to 0 indicate a high degree of agreement between participants, while higher values show a lesser degree of agreement. The formula used to calculate H scores is depicted below: *K* refers to the number of different names provided for each celebrity and *p*_*i*_ is the proportion of responses to each proper name. The three categories of naming failures (i.e., participants reported “do not know the celebrity”, “do not know his/her name” or a “ToT” state), that were included when calculating proper name agreement, were eliminated when calculating H.

$$H=\sum_{i=1}^{K}p_{i\;}{log}_{2}(1/p_{i})$$

Notice that, similar to Snodgrass and Vanderwart [8], participants from this study were allowed to produce the first name that came to mind. This procedure implied that participants were free to produce the first name/s, the second name/s or both. For this reason, more variability than in Bonin et al’s study was expected, and, consequently, we computed separate indices for the cases where the first name/s only, the second name/s only, or the complete names were produced.

Statistics showed different levels of proper name agreement whether the first name/s only (*M* = 0.22, *SD* = .29), the second name/s only (*M* = 0.19, *SD* = 0.20), or the complete names (*M* = 0.29, *SD* = 0.26) were produced [*F*(1,17) = 22.103, *p <* .0001, *η*^*2*^ = 0.56]; participants produced more first names than second names [*t*(17) = -2.987, *p* = .008, *d* = 0.70], and more complete names than first names [*t*(40) = -4.877, *p <* .0001, *d* = 0.76].

The three distributions for first, second, and complete names were positively skewed, indicating that more frequent instances had lower levels of PNA. The PNA values from this study were lower than those reported by Bonin et al. [16] [for the comparison between the conditions where the complete name was produced, *t*(189) = 12.05, *p <* .0001, *d* = 0.26], and the *H* scores showed a higher degree of disagreement [for the comparison between the conditions where the complete name was produced, *t*(131.110) = -4.980, *p <* .0001, *d* = 0.73]. However, in the current study the *H* statistics showed a positive asymmetry in all the conditions, indicating that the most frequent values had low scores in the three distributions.

Notice that the general low name agreement in the current study (see PNA and H values) is not easily explained by misidentification of facial features (i.e., naming Koffi Annan because his face can be confused with Morgan Freeman’s) but, as explained before, the instructions during the naming task could have favored the production of a wide range of correct instances for the same celebrity name (i.e., when presented the photograph of the ex-prime minister José Luis Rodríguez Zapatero, participants could say José Luis, Zapatero, José Luis Rodríguez, Rodríguez Zapatero, etc.). In consequence, name agreement in the current study could be located at the FRU level, when naming failures involved incorrect names (i.e., saying Morgan Freeman instead of Koffi Annan) or at a post-structural level when different tokens from the same celebrity name are produced (i.e., saying Zapatero or José Luis Rodríguez Zapatero). Therefore, for the sake of completeness, we estimated the percentage of participants who produced a correct name for each celebrity (percentage of responses), an average of all correct responses regardless of whether the first, the second, or the complete name were produced. Given that this variable comprises all the correct instances for a celebrity name, but not the incorrect ones, we considered that it could be more specific of the FRU level than the PNA or H indexes.

The mean percentage of correct identificatory responses was 46%, indicating that, on average, correct names were produced half of the times. We must note however that, in spite of this low mean value, the variability of the data was very high (*SD* = 34%), ranging from no correct naming at all for some celebrities to a 100% of correct responses when other celebrities were named. Given that Smith-Spark et al. [19] provided a measure of correctly generated written names, we compared both data sets. Results showed a higher number of correct responses in our study (*M* = 46.45, *SD* = 33.6 vs. *M* = 18.7, *SD* = 16.7), *t*(126.92) = 8.785, *p* < .0001, *d* = 1.3. It should be noted, however, that Smith-Spark et al. presented a larger number of celebrities, and it is likely that the low level of correct naming in their study was due to the lower familiarity of some of the celebrities.

Regarding the rating scales, we first analyzed face agreement scores. A high face agreement value could be indicative of a high degree of overlapping between the perceived face and the stored representation; in consequence, in terms of theoretical relevance, face agreement could also affect the FRU level [16]. Our data showed a high correspondence between the collected photographs and the participants’ mental image of the celebrities (*M* = 3.93; *SD* = 0.80). In this line, the negative asymmetry of the face agreement distribution indicated that higher values were more frequent. These face agreement scores were higher than the values obtained by Bonin et al. [*t*(218.350) = -2.924, *p* = .004, *d* = 0.39].

Similarly to face agreement, distinctiveness has also been proposed to affect the FRU level, facilitating face selection because the relative uniqueness of the stored FRU representation results in less competition with other FRUs [16,60]. Distinctiveness scores were generally high (*M* = 4.89, *SD* = 0.82), and when compared to the norms assembled by Bonin et al. [16] and by Smith-Spark et al. [19] significant differences were found [*F*(2,919) = 197.948, *p <* .0001, *η*^*2*^ = .302]. The study conducted by Bonin et al. showed the highest levels of distinctiveness (*M* = 5.41, *SD* = 1), while the lowest scores were obtained in the norms by Smith-Spark et al. (*M* = 3.67, *SD* = 1) [*t*(201.635) = 4.214, *p* < .0001, *d* = 0.57; *t*(799) = 16.677, *p* < .0001, d = 1.74; *t*(180.523) = 14.408, *p* < .0001, *d* = 1.2; comparisons between, Bonin et al. and our study, Bonin et al. and Smith-Spark et al., and our study and Smith-Spark et al. study, respectively].

The number of phonemes for each celebrity name has been considered an important determinant of naming speed (e.g., [61], for objects; Bonin et al. [16], for faces). Our results indicated that the mean of this variable was 10.03 (*SD* = 3.56). There were not differences between the data in our study and the data in the studies by Bonin et al. and by Smith-Spark et al. [*F*(2, 919) = 0.476, *p* = .62, *η*^*2*^ = .001].

The frequency with which we encounter a celebrity in the real word (subjective frequency) is also a relevant variable affecting face naming [62,35], and it has been proposed to operate at a phonological stage [12,63,64] or at the semantic-to-lexical level [65,66]. The mean statistic for subjective frequency was located at the once-per-month score (*M* = 3.16, *SD* = 0.84), results that did not differed from the data in Bonin et al. [16] [*t*(221) = -1.618, *p* = .107, *d* = 0.22].

Age of acquisition has traditionally been considered an important predictor of naming speed (e.g., [67–70]), although the locus of the effect is not totally clear. Similar to subjective frequency, some researchers localize AoA effects at the phonological level (e.g., [71–73]) while others place it at the semantic-to-lexical level [74,66]. In the face-naming field, a consistent AoA effect has been observed, with studies demonstrating faster naming times for faces encountered early in life [16,62]. Regarding the AoA scores obtained in the present study (*M* = 12.71, *SD* = 2.42), it is noticeable that, although we employed a data collection procedure (direct question) that was different from the one used by Bonin et al. (scale rating), the AoA means in both data sets were close to 12 years old. This similarity stands in contrast to the values reported by Smith-Spark et al. [19], where the mean AoA for the studied celebrities was between 25 and 35 years of age. A likely explanation of this disparity is that all the participants in the study by Smith-Spark et al. were over 40 years old, and their average age was 62 years.

ToTs are reported to be more frequent during face naming than during object naming [75], a finding traditionally attributed to the unique connections between person identity and the phonological representation [37]. For this reason, we collected ToTs in both the proper name agreement task and the response time task. Since results in both tasks were highly correlated (*r* = .69, *p* < .0001), similar to Bonin et al. [16], we selected the ToTs values collected during the proper name agreement task for the subsequent analyses. Participants rarely reported ToTs experiences (*M* = .10; positive asymmetry), in contrast with a higher frequency of ToTs in the Bonin et al. study [*t*(164.278) = 6.330, *p <* .0001, *d* = 0.87].

As described above, we also collected emotional scores for each celebrity using the Self-Assessment Manikin (SAM; [50,51]), obtaining indicators of valence (1-happy to 9-unhappy; *M* = 4.67, *SD* = 1.22), arousal (1-excited to 9-calm; *M* = 4.81, *SD* = 0.49), and dominance (1-controlled to 9-in-control; *M* = 5.48, *SD* = 0.35). Each dimension differed in their skew values, and the most frequent responses were closer to the calm, happy, and in-control states.

### Correlations

Table 3 contains the complete set of correlations among all the previously described variables. Subjective frequency was positively correlated with AoA, distinctiveness, face agreement, ToTs, number of phonemes, percentage of responses, and PNA. That is, celebrities who were more frequently encountered were perceived as more distinctive, were late-acquired, had a higher level of face-name agreement scores, were more easily named, presented higher name agreement scores (despite having longer names), and were more vulnerable to ToT states. In turn, distinctiveness was positively correlated with face agreement, percentage of responses, and PNA, and negatively with AoA and number of phonemes. This finding indicates that highly distinctive faces tended to be early-acquired, showed higher levels of face agreement scores, elicited more correct names with higher agreement scores, and had names that were shorter in length.

**Table 3:** Correlations.

	**AoA**	**distinctiveness**	**face agreement**	**subjective frequency**	**PNA**	**H**	**ToT**	**number of phonemes**	**percentage of responses**	**valence**	**arousal**
**distinctiveness**	-.37**										
**face agreement**	-.12	.59**									
**subjective frequency**	.43**	.18*	.51**								
**PNA**	.11	.23*	.53**	.50**							
**H**	-.06	.17	.1	.14	-.27*						
**ToT**	.22*	.08	.37**	.29**	.02	.11					
**number of phonemes**	.15	-.2*	.08	.28**	.18	-.16	.13				
**percentage of responses**	-.16	.54**	.75**	.49**	.71**	-.00	.13	-.10			
**valence**	.11	-.05	-.16	.14	-.09	-.01	-.15	.11	-.15		
**arousal**	.13	-.49**	-.35**	-.02	-.19	-.04	-.12	.20*	-.33**	.45**	
**dominance**	.11	.11	.23*	.29**	.24*	.06	.16	.18*	.28**	.38**	.22*

Note:

*p< .05,

** p < .01

Face agreement was positively correlated with ToTs, PNA, and percentage of responses. This is, faces with higher level of face-name agreement scores, showed also more name agreement and generated more correct responses and ToT experiences. Moreover, AoA was positively correlated with ToTs, an indication that later-acquired celebrities elicited more ToT states. As expected, PNA and H statistics showed a negative correlation (celebrities with higher proper-name agreement had lower H scores). Notice that the correlation between PNA and H scores, although significant (*r* = -.26, *p* = .01), was numerically lower than the values previously obtained in other studies. For example, the correlation observed in Snodgrass and Vanderwart [8] was almost perfect (*r* = -.94). A possible reason for this discrepancy could be the relatively high number of response failures observed in the current study. Thus, as explained before, in the PNA agreement computation, response failures are taken into account, while these data were eliminated for H statistic. This difference in the computation of these two indices should not affect the correlation values when the number of failures is low (such as in Snodgrass & Vanderwart’s study), but, in the cases where the number of response failures increases (like in the current study), the correlation values are expected to decrease. In fact, if we eliminate the response failures in the PNA computation, the correlation with H scores is also almost perfect (*r* = -.93, *p <* .0001).

Regarding the relationship between the emotional dimensions and the rest of the variables, we observed a positive correlation between dominance, valence, arousal, face agreement, frequency, percentage of responses, PNA, and number of phonemes. That is, celebrities that elicit more dominant (in-control) states, tend to be associated to less happy and calmer states. In addition, these celebrities are judged to be more frequently encountered, showed higher levels of face agreement scores, and elicited more name responses with higher name agreement that were longer in length. There were also significant correlations between arousal, valence, and number of phonemes (positive), and arousal, distinctiveness, face agreement, and percentage of responses (negative). In this line, happier and more arousing states are associated with shorter names. In addition, these states were elicited when celebrities were more distinctive, had higher face agreement scores, and elicited more correct names.

### Naming times

The same filters and trimming procedure employed in Bonin et al. [16] were used in the present study. As a result, data from one subject with a low number of correct responses (only 15%) were deleted, and item naming times that exceeded two standard deviations from the mean were also eliminated.

Following Bonin et al. [16], AoA, distinctiveness, face agreement, subjective frequency, number of phonemes, and PNA, were submitted to a step-wise regression analysis with RTs as the dependent variable. In addition, we also included the variable percentage of responses and valence, arousal, and dominance, as possible predictors. Results indicated that only the variable percentage of responses entered in the model (see Table 4). Similar to Bonin et al. [16], we also rerun this analysis with only those cases where PNA scores were above the median. Now, the variables percentage of responses and dominance entered in the model. When PNA was deleted from the analysis, again percentage of responses was a significant predictor. Given that percentage of responses seemed to be a more relevant variable than PNA in this dataset, we rerun the step-wise regression analysis with only those cases where the percentage of responses was higher than the median. With this data set, the variables percentage of responses and dominance entered in the model. When percentage of responses was eliminated from the model, distinctiveness was the only variable to enter in the model.

**Table 4:** Step-wise regression with RTs as dependent variable.

Variables	B	SE	t	Sig.	R2
**Percentage**	-267.748	74.876	-3.576	.001	.139
	**PNA not included**				
**Percentage**	-274.806	68.082	-4.036	.000	.138
	**PNA > Median**				
**Percentage**	-550.632	111.160	-4.954	.000	.449
**Dominance**	-139.624	58.755	-2.376	.022	
	**percentage not included**				
**Distinctiveness**	-97.702	31.229	-3.129	.002	.110
	**percentage > Median**				
**Percentage**	-433.472	136.527	-3.175	.003	.223
**Dominance**	-116.694	56.192	-2.077	.044	

### ToTs

The predictors AoA, distinctiveness, valence, arousal, dominance, face agreement, subjective frequency, number of phonemes, PNA, and percentage of responses were submitted to a step-wise regression analysis, with number of ToTs as the dependent variable. The results (see Table 5) showed that both face agreement and percentage of responses predicted ToTs. When the same analysis was performed with PNA scores above the median, again the variables face agreement and percentage of responses entered in the model. When PNA was eliminated, face agreement, percentage of responses, and AoA predicted ToTs. Given that percentage of responses is also a consistent predictor when studied ToTs, we again rerun the analysis only with the values of percentage of responses higher than the median. The variables percentage of responses and face agreement predicted the ToTs. When percentage of responses was eliminated, only face agreement entered in the model.

**Table 5:** Step-wise regression with ToTs as dependent variable.

Variables	B	SE	T	Sig.	R2
**face agreement**	0.074	0.020	3.6641	.001	.183
**Percentage**	-0.108	0.043	-2.4967	.015	
	**PNA not included**				
**face agreement**	0.058	0.013	4.376	.000	.243
**Percentage**	-0.079	0.033	-2.37	.020	
**AoA**	0.007	0.003	2.353	.021	
	**PNA >Median**				
**Percentage**	-0.441	.053	-8.271	.000	.688
**face agreement**	0.105	.031	3.378	.002	
	**percentage not included**				
**face agreement**	.038	.015	2.579	.012	.098
	**percentage > Median**				
**Percentage**	-.546	.055	-9.860	.000	.763
**face agreement**	.152	.031	4.886	.000	

In general, the percentage of correct responses was a relevant predictor in both ToTs and RTs. This result contrasts to previous face naming [16] and object naming studies (e.g., [59]) in which name agreement has consistently been shown to predict RTs. A possible reason why we failed to observe a name agreement effect could be the higher variability allowed by the face naming task in our study. We did find, however, that percentage of responses, that we located at the FRU level, was a significant predictor of RTs. Smith-Spark et al. [19] also observed that this variable was relevant in celebrity name production, and, although they did not collect RTs nor carried out regression analyses, they found that percentage of responses was significantly correlated with other relevant dimensions, such as AoA and distinctiveness. The relevance of the percentage of correct responses in face naming is consistent with the most accepted models of face processing (e.g., [39,40]). From this view, faces with higher levels of correct responses could be more easily identified at the FRU level, in consequence, the naming times would be faster and the number of incorrect responses would decrease. In addition, dominance was a significant predictor of RTs when cases above the PNA median were selected, with more dominant states associated to faster RTs. This is an interesting result, given that most normative studies have focused on valence and arousal (e.g., [76–80]), with words in several languages; or [81,82], with visual stimuli), and it points to the potential relevance of the effects of dominance when face naming is the focus of interest (see for example, [83], for a normative study on dominance for unfamiliar faces).

Face agreement also seemed to play a role in ToT states, with less ToTs produced when face agreement had higher values. This result is consistent with the findings by Bonin et al. [16], and theoretically consistent with FRU-level involvement [39,40], since a high agreement score would entail, first, a high degree of overlapping between the perceived face and the stored representation and, second, an easier face recognition. In a similar vein, AoA significantly predicted ToTs when PNA was not included, but not in the rest of the analyses. These finding contrasted with Bonin et al.’s data, where AoA was a significant predictor of both RTs and ToT. We must notice, however, that AoA effects can be complex, as evidenced in word-based studies (e.g., [68,84]), and the results of the present study might well constitute relevant evidence for current debates on the issue.

## Conclusions

Normative ratings for a set of 118 celebrities in Spain were obtained from a large sample of college students. Specifically, AoA scores, distinctiveness, emotional ratings (valence, arousal, and dominance), face agreement, subjective frequency, number of phonemes, proper name agreement (PNA), H, percentage of correct responses, ToTs, and response times (RT) were collected for each celebrity.

The psychometric properties associated to the current norms can be considered appropriate, as these norms showed a general high reliability (average of .83) and a high validity (HA average of .81).

Several general results are worth noticing. The percentage of correct responses (but not proper name agreement) was consistently a significant predictor of response times (RTs) and ToTs. This result is not surprising given the low level of proper name agreement in the current norms. Face agreement and AoA (only when PNA is eliminated) seemed also to predict less ToT states, and an emotional variable, i.e., dominance, predicted RTs (when PNA scores above the median were selected). These results point to the convenience of collecting (in celebrity normative studies) and/or controlling (in experimental research) both psycholinguistic (i.e., face-agreement, AoA) and emotional variables (i.e., dominance scores). In fact, the relevance of the emotional correlates for experimental stimuli is mainly noticeable when focused on the large number of normative studies that provide emotional ratings for different materials. As an example, only the group headed by Margaret Bradley and Peter Lang have collected emotional ratings for words (Affective Norms for English Words, ANEW; [85]), digital sounds (International Affective Digital Sounds, IADS; [86]), colored photographs (International Affective Picture System, IAPS; [87]), or brief texts (Affective Norms for English Texts, ANET; [88]). In this vein, the current norms extend the study of the emotional dimensions to a set of materials where emotions are assumed to play a relevant role, this is, familiar faces (e.g., [47]).

These celebrity norms contain a number of indices that have also been collected in object naming studies (i.e., AoA, PNA, distinctiveness, etc.). We must mention that, although these data could allow to establish comparisons between face and object naming processing, the possible conclusions should be taken with caution, given that it is not totally clear if the mentioned variables refer to the same construct in the face and object fields (see for example [89], for a discussion on the problems of the analogy between words and faces).

Notice that the results of these rating tasks could be considered image specific more than celebrity specific, more so in the tasks where a photograph of the celebrity is presented (face-name agreement task, distinctiveness task, and affective evaluation task) than when the celebrity name appears in isolation (subject frequency task and age of acquisition task). But in any case, the results obtained in the face-name agreement task clearly indicated that, in general, there is a high correspondence between the collected photographs and the participants’ mental image of the celebrities.

In conclusion, these norms are expected to be a valuable tool in many cognitive areas such as psycholinguistics (e.g., [22,26,28]), memory (e.g., [90]), face processing (e.g., [36–39]), healthy and pathological aging [20,29–33,91,92], or studies about emotion [48,93]. In spite of the usefulness of this type of norms in a wide range of areas, we must notice a series of limitations of the specific set of celebrities produced in each normative study. First, these norms are strongly restricted to a particular geographic location and socio-cultural context, as mentioned in the introduction. In addition, although there were coincidences between the values obtained in our study and data from previous norms, we have also observed noticeable differences (thoroughly described in the Results section) that would support the usefulness of collecting normative data for specific contexts. Moreover, even in the same country the exemplars in some categories of celebrities could be relatively stable (e.g., royalty members, painters, historical figures, etc.) while in other categories celebrity status could be sub-culture specific, with some personalities highly familiar to some people, and completely unfamiliar to others (e.g., football players, basketball players, formula 1 drivers, etc.). In an attempt to minimize this limitation, this study offers an extended set of celebrities from a wide range of categories. It is also the case that the characteristics of the population, e.g., the age of the participants (from 40 to 91 years old in Smith-Spark’s study, and undergraduate students in Bonin et al’s study) could contribute to the sometimes varied results obtained in the current and other available celebrity norms. Because different cohorts are likely to be exposed to different celebrities, the use of these norms, built from a college student sample of young adults, with other age populations should be made with caution. In addition, the passage of time can be a significant modulating factor. While this could be an issue for almost any normative study, the transient nature of fame can more quickly determine which celebrities are to be of relevance in a given study. For example, Smith-Spark et al. [19] noted that the recency of the death in 2005 of Pope John Paul II could have increased his rated familiarity in their norms. Although our normative study includes a number of well-known celebrities (charismatic actors and actresses, internationally acclaimed sportmen and sportwomen, historical figures, etc.) that could be considered relatively stable over time, the fame of the celebrities in other categories can be considered more transient (e.g., gossip-show celebrities). In consequence, we need to be cautious with employing these materials over a long period of time. If the need for geographical and contextual specificity were the motive for the development of the current study, the need for periodical revisions, generational adjustments, and cultural sensitivity point to future productive efforts in this area of normative studies.

## Supporting information

S1 Table Stimuli and data. (XLSX)
